# Detection and quantification of novel C‐terminal TDP‐43 fragments in ALS‐TDP

**DOI:** 10.1111/bpa.12923

**Published:** 2021-01-29

**Authors:** Emily Feneberg, Philip D. Charles, Mattéa J. Finelli, Connor Scott, Benedikt M. Kessler, Roman Fischer, Olaf Ansorge, Elizabeth Gray, Kevin Talbot, Martin R. Turner

**Affiliations:** ^1^ Nuffield Department of Clinical Neurosciences John Radcliffe Hospital University of Oxford Oxford UK; ^2^ Nuffield Department of Medicine Centre for Medicines Discovery Target Discovery Institute University of Oxford Headington UK

**Keywords:** Alzheimer’s disease, amyotrophic lateral sclerosis, LATE, proteomic biomarker, TDP‐43

## Abstract

The pathological hallmark of amyotrophic lateral sclerosis (ALS) is the presence of cytoplasmic inclusions, containing C‐terminal fragments of the protein TDP‐43. Here, we tested the hypothesis that highly sensitive mass spectrometry with parallel reaction monitoring (MS‐PRM) can generate a high‐resolution map of pathological TDP‐43 peptide ratios to form the basis for quantitation of abnormal C‐terminal TDP‐43 fragment enrichment. Human cortex and spinal cord, microscopically staged for the presence of p‐TDP‐43, p‐tau, alpha‐synuclein, and beta‐amyloid pathology, were biochemically fractionated and analyzed by immunoblot and MS for the detection of full‐length and truncated (disease‐specific) TDP‐43 peptides. This informed the synthesis of heavy isotope‐labeled peptides for absolute quantification of TDP‐43 by MS‐PRM across 16 ALS, 8 Parkinson’s, 8 Alzheimer’s disease, and 8 aged control cases. We confirmed by immunoblot the previously described enrichment of pathological C‐terminal fragments in ALS‐TDP urea fractions. Subsequent MS analysis resolved specific TDP‐43 N‐ and C‐terminal peptides, including a novel N‐terminal truncation site‐specific peptide. Absolute quantification of peptides by MS‐PRM showed an increased C:N‐terminal TDP‐43 peptide ratio in ALS‐TDP brain compared to normal and disease controls. A C:N‐terminal ratio >1.5 discriminated ALS from controls with a sensitivity of 100% (CI 79.6–100) and specificity of 100% (CI 68–100), and from Parkinson’s and Alzheimer’s disease with a sensitivity of 93% (CI 70–100) and specificity of 100% (CI 68–100). N‐terminal truncation site‐specific peptides were increased in ALS in line with C‐terminal fragment enrichment, but were also found in a proportion of Alzheimer cases with normal C:N‐terminal ratio but coexistent limbic TDP‐43 neuropathological changes. In conclusion this is a novel, sensitive, and specific method to quantify the enrichment of pathological TDP‐43 fragments in human brain, which could form the basis for an antibody‐free assay. Our methodology has the potential to help clarify if specific pathological TDP‐43 peptide signatures are associated with primary or secondary TDP‐43 proteinopathies.

## Graphical abstract

The pathological hallmark of amyotrophic lateral sclerosis (ALS) is the presence of cytoplasmic inclusions, containing C‐terminal fragments of the protein TDP‐43. Here, we used a high‐resolution map of TDP‐43 peptides to form the basis for absolute quantitation of abnormal C‐terminal TDP‐43 fragment enrichment. The C:N‐terminal peptide ratio is increased in ALS brains and N‐terminal truncation‐specific peptides confirm the pathological cleavage of TDP‐43 in ALS and surprisingly AD.

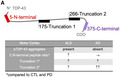

## 1 INTRODUCTION

The presence of ubiquitylated cytoplasmic inclusions of the 43 kDa transactive region DNA‐binding protein, TDP‐43, is the neuropathological hallmark of the neurodegenerative disorder amyotrophic lateral sclerosis (ALS) in 97% of all cases and 50% of cases of frontotemporal dementia (FTD) (1, 2). TDP‐43 histopathology also defines “limbic‐predominant age‐related TDP‐43 encephalopathy (LATE)” which may coexist with Alzheimer’s disease (AD) neuropathological change (3). However, the principal neuroanatomical distribution of microscopic TDP‐43 pathology in LATE is distinct from that of classical ALS‐TDP. The neuropathological features of TDP‐43 proteinopathy in ALS and FTD include nuclear to cytoplasmic mislocalization, posttranslational modifications such as ubiquitylation and phosphorylation, aggregation, and N‐terminal truncated C‐terminal TDP‐43 fragments (CTFs) (1, 4). When extracting the insoluble protein fraction from *post mortem* tissue with TDP‐43 proteinopathy, a pathological signature can be detected by immunoblotting, which is characterized by a 45 kDa full‐length protein band (the higher molecular mass smear representing posttranslational modifications), and lower molecular weight bands (20–35 kDa), which have been identified as CTFs (1, 5). Although TDP‐43 neuropathology has been well defined using immunohistochemistry (6, 7), biochemical detection of the pathological TDP‐43 forms has so far been limited to semi‐quantitative detection by immunoblotting of the insoluble protein fractions from *post mortem* tissue and has not yet been reproduced in biofluids (8). Studies using antibodies against TDP‐43 and its phosphorylated form to absolutely quantify pathological TDP‐43 by ELISA in cerebrospinal fluid (CSF) or serum have provided inconsistent findings and antibodies have failed to detect phosphorylated TDP‐43 or CTFs in lumbar CSF (9, 10). Limitations are the significantly lower amounts of pathological TDP‐43 present in a complex matrix such as CSF or serum, nonspecific binding of commercial antibodies to immunoglobulins, and the limited availability of antibodies that specifically detect pathological, brain‐derived TDP‐43, including discriminating normal full‐length TDP‐43 from pathological truncated CTFs (11, 12, 13).

We aimed to develop an antibody‐independent method to biochemically detect CTFs by mass spectrometry to enable the absolute quantification of pathology. Detecting specific C‐ and N‐terminal TDP‐43 peptides allowed us to measure the pathological accumulation of C‐terminal TDP‐43 relative to its N‐terminal end in urea fractions extracted from TDP‐43 pathology positive ALS *post mortem* tissue. The detection of additional N‐terminal truncation site‐specific peptides was used to quantify the process of pathological cleavage of TDP‐43 into N‐terminal truncated CTFs. This method specifically discriminated ALS from other neurodegenerative diseases such as Parkinson’s disease (PD) and AD. Interestingly, by this method we were able to identify some cases of AD with coexistent LATE neuropathological change (LATE‐NC); however, further work is required to establish whether our method is suitable for the identification of disease‐specific “TDP‐43 strain signatures” associated with the spectrum of primary and secondary TDP‐43 proteinopathies.

## 2 MATERIALS AND METHODS

### 2.1 Human samples

Primary motor cortex (BA4) or, if unavailable, dorsolateral prefrontal cortex (BA9/46) and spinal cords from individuals diagnosed with ALS, PD or AD, and from normal controls (CTL) were obtained from the Oxford Brain Bank. Donors had provided written informed consent for brain donation and the use of the material and clinical information for research purposes under Research Ethics Committee approval (REC 15/SC/0639, HTA license 12217).

### 2.2 Clinical characteristics

For several ALS cases severity of physical symptoms was classified as per the revised ALS Functional Rating Scale (ALSFRS‐R) with a maximum of 48 points. Here, lower values represent a more severe disease stage (14) and the progression rate was calculated as 48 minus the ALSFRS‐R score at sampling divided by the disease duration from onset of symptoms and sampling (point/month) (15). The region of onset was defined as where the first sign of disease was reported (spinal or bulbar) and the diagnostic certainty reported according to the revised El Escorial Criteria (16). *Post mortem* delay was calculated in hours from time of death to fixation and the age at death was recorded. Genetic testing was only conducted in the presence of a clear family history.

### 2.3 Immunohistochemistry for microscopic analysis of neurodegenerative proteinopathies

All cases were formally assessed for microscopically visible protein aggregates using standard criteria used in international brain banking (PMC3266529). In brief, formalin‐fixed paraffin‐embedded sections (six microns) were stained with the following primary antibodies (source, dilution and antigen retrieval method in parentheses): phosphorylated TDP‐43 (pTDP‐43) that targets pS409/410 (Cosmobio, TIP‐PTD‐M01, 1:20,000, autoclave—citrate buffer); Anti‐ß‐Amyloid (4G8—Biolegend, 1;24,000, formic acid); AT8 (Innogenetics, 1:1500, none); and Purified Mouse Anti‐alpha‐Synuclein (BD Laboratories, 1:1000, formic acid). All were developed with the DAKO Envision Kit and 3,3‐diaminobenzidine tetrahydrochloride (DAB) as chromogen. Sections were examined using an Olympus BX50 microscope with an attached Olympus digital camera (for photography).

### 2.4 Extraction of soluble and insoluble protein fractions from tissue

Fractionation of tissue was performed as described by Neumann and colleagues (1), with the following modifications. Frozen tissues from cortex and spinal cord of all groups of subjects were homogenized in 1:1 volume: tissue weight low‐salt homogenization‐solubilization (LS) buffer (10 mM Tris pH 8.0 (Invitrogen), 5 mM EDTA (Invitrogen), 1 mM dithiothreitol (Alfa Alsar) supplemented with protease and phosphatase inhibitors (Thermo Fisher), 0.2 mM Sodium orthovanadate, and 0.3 Unit/µl benzonase (Merck)). Samples were homogenized using steel beads in a TissueLyser system (Qiagen) and centrifuged at 21,000 g for 30 min at 4°C. The pellets were homogenized in Triton‐X (TX) buffer (1% Triton X‐100, 0.5 M NaCl in LS buffer). Protein amount was determined by BCA assay (Thermo Fisher) and all samples were equalized to 20 mg of total protein in a final volume of 3 ml Triton‐X buffer. Tissue homogenates were centrifuged at 180,000 g for 30 min at 4°C in thin‐wall polypropylene tubes (Beckman Coulter) in swinging buckets in an Optima MAX‐XP ultracentrifuge (Beckman Coulter). Supernatants, corresponding to the soluble proteins, were saved as the Triton‐X fraction (TX). Pellets were washed in TX buffer supplemented with 30% of sucrose (Merck). Pellets were subsequently resuspended in sarkosyl buffer (1% N‐lauroyl‐sarcosine (Merck), 0.5 M NaCl in LS buffer), briefly sonicated, and incubated at 37°C on a shaker (160 rpm) for 30 min followed by centrifugation at 180,000 g for 30 min at 22°C. Supernatants were saved as the sarkosyl fraction (SF), while the pellets were resuspended in urea buffer (7 M urea (Merck), 2M Thiourea (Merck), 4% CHAPS (Thermo Fisher), and 30 mM Tris pH 8 (Invitrogen)) supplemented with protease and phosphatase inhibitors (Thermo Scientific). Samples were sonicated and centrifuged for 30 min at 21,000 g at room temperature. The supernatant, corresponding to the insoluble fraction, was saved as the urea‐soluble fraction and frozen at −80°C prior to use. Four samples randomly chosen from each diagnostic group were fractionated together.

### 2.5 Immunoblotting

The urea fractions were diluted in LDS sample buffer (Thermo Fisher) supplemented with reducing agents (Thermo Fisher) and boiled for 5 min. Proteins were separated on 10% NuPAGE pre‐cast Bis‐Tris gels (Thermo Fisher), transferred using an iBlot2 apparatus (Thermo Fisher). Membranes were Ponceau‐stained, then blocked using protein free Blocking buffer (Thermo Fisher), and probed overnight at 4°C with a polyclonal TDP‐43 C‐terminus antibody recognizing TDP‐43 at epitopes from amino acid 260 onwards (Proteintech, 12892‐1‐AP) or phosphorylated TDP‐43 at S409/410 (Proteintech, 22309‐1‐AP) and 2 h at room temperature with secondary antibody rabbit HRP. Antibody signal was detected with ECL reagent (GE Healthcare) using a ChemiDoc MP Imaging System (Bio‐Rad). For interpretation of the immunoblots the ratios of the intensities of the CTFs over full‐length TDP‐43 were compared.

### 2.6 Silver staining

Total protein amounts were visualized by silver staining using a ProteoSilver Silver Stain kit (Merck) as per the manufacturer’s protocol and using solutions provided in the kit. Briefly, after electrophoresis, NuPAGE Bis‐tris gels were fixed overnight at room temperature in fixing solution (10% acetic acid (Millipore) and 50% Ethanol (VWR) in MilliQ water). The gels were consecutively washed for 10 min in 30% Ethanol, MilliQ water, sensitizer solution, and silver solution. Staining was visualized by incubating with developer solution for 30–70 s, the reaction was stopped by addition of ProteoSilver Stop solution and washing with MilliQ water for 15 min.

### 2.7 Sample preparation for mass spectrometry

Urea fractions were thawed on ice and used for digestion. For in‐gel digestion gel bands were excised with a scalpel after electrophoresis and gel pieces rinsed in 200 μl wash solution (50% methanol and 5% acetic acid in MilliQ water). Two‐hundred microliters of acetonitrile was added to dehydrate the gel pieces prior to reduction with 5 mM DTT and alkylation with 20 mM IAA. After dehydration samples were first rehydrated in 200 μl of 100 mM ammonium bicarbonate and then, in 30 µl of 200 ng/µl chymotrypsin (Promega) in 50 mM ammonium bicarbonate and incubated overnight. Fifty microliters of 50 mM ammonium bicarbonate buffer was added to the samples, 50 µl of extraction buffer 1 (50% acetonitrile and 5% formic acid in MilliQ water) and 50 µl of extraction buffer 2 (85% acetonitrile and 5% formic acid in MilliQ water) were added and supernatant with eluted peptides collected. For in‐solution digestion samples were reduced with 5 mM of dithiothreitol (Alfa Alsar) followed by alkylation with 20 mM IAA. Samples were subsequently precipitated by methanol–chloroform extraction. Pellets were fully resuspended by addition of 6 M urea buffer (Merck) and brief sonication. Samples were reduced to a final concentration of 1 M Urea by adding 50 mM TEAB buffer. The samples were incubated with 2 μg trypsin/chymotrypsin (Promega) at 37°C/25°C on a shaker for 14–16 h. The reaction was stopped by addition of formic acid (Millipore). Samples were desalted using SOLA SPE columns (Thermo Fisher). All in‐solution and in‐gel digested samples were dried in a speed‐vac (Thermo Scientific) and pellets were resuspended in 2% acetonitrile with 0.1% formic acid diluted in MilliQ water.

### 2.8 Discovery proteomics

Peptides from in‐solution and in‐gel digestion were analyzed by nano ultra‐high‐performance liquid chromatography (nano‐UPLC) tandem mass spectrometry (MS/MS) using a Dionex Ultimate 3000 nano‐UPLC, (Thermo Fisher) coupled to an Orbitrap Fusion Lumos Tribrid mass spectrometer (Thermo Fisher). Data were acquired in data‐dependent mode with a resolution of 120,000 full width half maximum at m/z 200 in the survey scan (375–1500 m/z) and with EASY‐IC using the reagent ion source (202 m/z) for internal calibration. MS/MS spectra were acquired after precursor isolation in the quadrupole with an isolation window of 1.2 Th, dynamic precursor selection (top speed mode) with a 3‐s fixed duty cycle time, and 60‐s dynamic precursor exclusion. Isolated precursor ions were fragmented by CID with a Normalized Collision Energy of 35%. Parallelization was enabled and MS/MS spectra were acquired in the linear ion trap for up to 250 ms with an ion target of 4000 in rapid scan mode. Raw MS data were analyzed using Progenesis QI for Proteomics v3.0 (Nonlinear Dynamics). MS/MS spectra were searched against the UniProt Homo Sapiens Reference proteome (retrieved 15/11/2016) using PEAKS with precursor mass tolerance of 10 ppm and a fragment ion tolerance of 0.5 Da. Carbamidomethylation of Cysteines was defined as a fixed modification, deamidation of Asparagine and Glutamine, and oxidation of Methionine as variable modifications. Peptides scoring ≥20 and FDR <1% were imported into Progenesis QIP.

### 2.9 Truncation site‐specific peptide identification

As previously described, in‐gel digestion of low molecular weight bands (20–28 kDa) of ALS urea fractions were used to identify the truncation site‐specific TDP‐43 peptides (17). These peptides include one cleavage site independent of the enzyme used for digestion (giving a nonspecific digestion pattern), which suggests that cleavage has occurred endogenously prior to digestion. N‐terminal nonspecific peptides have an N‐terminal truncation site and cover an amino acid sequence toward the C‐terminus of TDP‐43, therefore, representing a specific truncation site of C‐terminal low molecular weight fragments. The Truncation 2 peptide contained an additional chymotryptic cleavage site after the position 5 leucine residue, therefore, the endogenous peptide is only produced by (relatively less frequent) incomplete digestion with chymotrypsin; a so‐called “missed cleavage.” The yield of the missed cleavage containing peptide from digestion could be assumed to be substantial since the peptide had been observed in the discovery experiment, so the peptide was explored despite this issue. Also, there was a relatively lower yield of this peptide after digestion in comparison to the other peptides, the mass spectrometer acquisition method was adapted to allow for this. The statistical analysis assumes that there was no significant variation in the site‐specific missed cleavage rate of chymotrypsin (and, therefore, final peptide yield) between samples.

### 2.10 Absolute quantification by parallel reaction monitoring

For the measurement of endogenous (light) TDP‐43 peptides by mass spectrometry with parallel reaction monitoring (MS‐PRM), C‐ and N‐terminal and truncation site‐specific peptides detected from the discovery experiments and a previously reported sequence (17) were used to generate heavy isotope‐labeled (heavy) peptides. Heavy peptides were commercially synthesized using AQUA‐grade (Thermo Fisher). One microliter of chymotrypsin‐digested and desalted samples were injected into the mass spectrometer. A time schedule targeted MS/MS method was used with peptide‐specific parameters as described in Table 2. Ten fmol of the heavy peptides listed was injected and, as technical quality check, pools of samples for each group were prepared and loaded every 10th run to determine the reproducibility of the assay. Samples were randomized per diagnostic group and run in ascending order according to TDP‐43 pathology (Ctrl, PD, AD, and ALS). MS data processing was conducted by an independent investigator blinded for diagnoses using the software packages Skyline and R. Briefly, Skyline was used to export ion chromatogram data for the light and heavy versions of each peptide in each analysis run as a text table that was then analyzed with an R script. The elution peaks of each light and heavy peptide pair were located in each sample. Pairs in each sample were filtered to remove those with insufficient signal for quantitation (minimum two monitorable fragment ion values measured over at least three collected spectra) or poor‐quality data (Pearson correlation between light and heavy chromatogram data less than 0.5). Elution peak boundaries were assigned at the point where the heavy peptide signal dipped below 1% of its maximum intensity in that sample. Finally, a light‐heavy ratio was calculated for each peptide by finding the gradient of a fitted simple linear model with no intercept (L = mH) to the light and heavy data points at each time point.

### 2.11 Statistical analysis

For interpretation of the PRM results absolute abundances of the light peptides (log_10_ ratio (light:heavy peptide)) were compared across diagnostic groups. The C:N‐terminal peptide ratio was calculated by dividing non‐log absolute abundances of the light peptides. All significant differences were tested comparing ALS to all other diagnostic groups using one‐way ANOVA with Dunnett’s multiple comparison test. *p*‐values <0.05 were considered as statistically significant. Standard measures of diagnostic test validity were performed on non‐log abundances of the light peptides. Sensitivity, specificity, and predictive values accompanied by their confidence interval (CI) stated at the 95% level were calculated for varying peptide cut‐off levels. The optimal cut‐off level for dichotomizing values was selected as the situation maximizing the Youden index (18). The receiver operating characteristics (ROC) curve is used for a graphical visualization of the impact of the variation in the cut‐off values.

## 3 RESULTS

### 3.1 *Post mortem* characterisation

All clinical diagnoses were neuropathologically confirmed and the characteristic neuropathology of each diagnostic group used for the final MS‐PRM analysis is shown in Figure S1. TDP‐43 neuropathology was qualitatively assessed using a phospho‐TDP‐43 antibody for the determination of typical inclusion pathology. The tissue subjected to MS‐PRM for the absolute quantification of peptides across diagnostic groups is presented in Table 1. The brain regions selected were motor cortex (region of the “hand knob”) or dorsolateral prefrontal cortex. The AD group was significantly older than CTL, ALS, and PD. *Post mortem* time from death to fixation was the same between groups (*p* = 0.6). Clinical characteristics of patients are given in Table S1. ALS patients, who donated tissue for this study, reported no family history and were apparently sporadic ALS cases.

**TABLE 1:** Characteristics of the *post mortem* tissue cohort

Diagnostic groups	Cortex, n (motor/frontal)	Spinal cord, n (thoracic/cervical/lumbar)	Mean (range) age (years)	Gender (f/m)
CTL	8 (8/0)	8 (7/1/0)	65.9 (38–89)	4/4
PD	8 (5/3)	6 (6/0/0)	76.9 (69–80)	1/7
AD	8 (8/0)	8 (8/0/0)	84.3*** (78–93)	4/4
ALS	16 (13/3)	16 (12/3/1)	65.6 (49–86)	6/10

Abbreviations: f, female, m, male; n, number.

*** Increased compared to all other groups (*p* < 0.001).

### 3.2 Discovery of TDP‐43‐derived peptides in brain urea fractions

**FIGURE 1:**
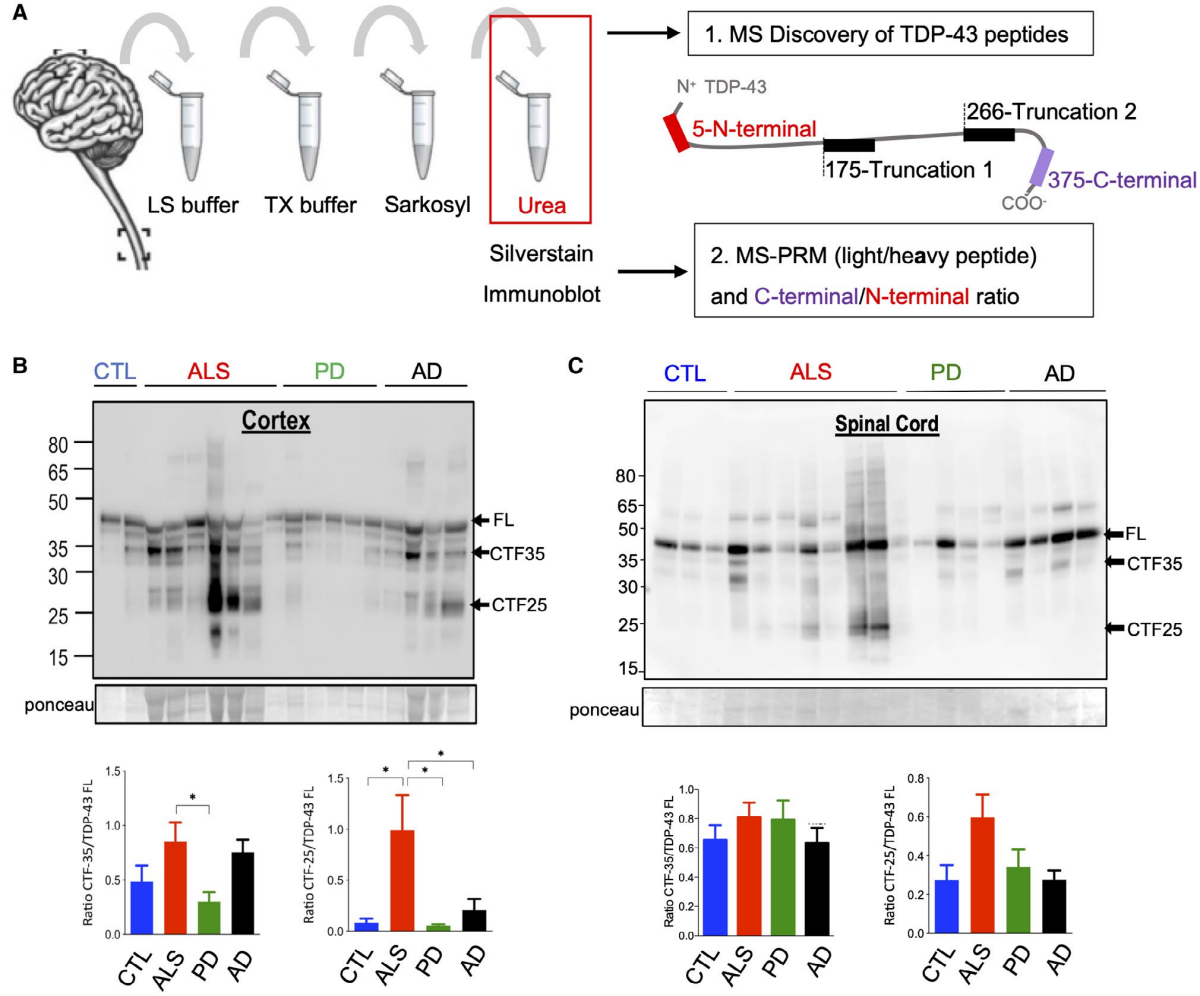
Urea fractionation of brain tissue for the enrichment of pathological TDP‐43. (A) Schematic of sample processing and TDP‐43 peptide identification. Given are the peptides and their initial amino acid within the full‐length TDP‐43 protein. (B and C) Representative immunoblots with an anti‐C‐terminal TDP‐43 antibody recognizing full‐length (FL) TDP‐43 at 43 kDa and smaller C‐terminal fragments (CTFs) of the insoluble (urea) protein fractions from MS‐PRM tissue cohort. (B) Cortex: Quantification of immunoreactive bands at 43 kDa, 35 kDa and 25 kDa demonstrated that the CTF‐35:FL TDP‐43 ratio in ALS was only increased when compared to PD (*p* = 0.04). CTF‐25:FL TDP‐43 ratio in ALS was increased compared to CTL, PD, and AD (*p* = 0.03, *p* = 0.02, and *p* = 0.048). (C) Spinal cord: Immunoreactive bands showed unaltered CTF‐35:FL and CTF‐25:FL TDP‐43 ratios in ALS compared to CTL, PD, and AD (*p* = 0.08, *p* = 0.17, and *p* = 0.053). Number of cases: 8 CTL, 8 PD, 8 AD, and 15 ALS. One‐way ANOVA with Dunnett’s multiple comparison test

First, we used LC–MS/MS analysis to detect unique peptides for the absolute quantification of TDP‐43 (Figure 1A). In‐solution digestion of two ALS and two CTL brain urea fractions revealed enzyme‐specific TDP‐43 peptides at the N‐terminal and C‐terminal end of TDP‐43 (Table S2). Chymotrypsin digestion achieved the best peptide coverage of TDP‐43 at its C‐terminus to allow for the subsequent detection of CTFs. By in‐gel digestion of low molecular weight bands (23–28 kDa) of two ALS brain urea fractions we were able to detect truncation site‐specific peptides that were semi‐specific for the enzyme chymotrypsin (Table S3). This included a novel N‐terminal truncation at amino acid 266 of TDP‐43 representing a C‐terminal TDP‐43 fragment detected at the size of 25 kDa (Truncation 2). A truncation site‐specific peptide (truncation 1) closer to the N‐terminus of TDP‐43 (aa175) was previously reported by Kametani et al., but not detected in our discovery analysis (17).

For absolute quantification of endogenous (light) peptides by MS‐PRM the corresponding heavy isotope‐labeled analogs of a list of tryptic and chymotryptic TDP‐43 peptides were generated (Table S4) and tested for technical reliability. For the final MS‐PRM analysis four chymotryptic peptides located at the N‐and C‐terminus of TDP‐43 including truncation 1 and 2 peptides were selected. The PRM properties of all four heavy peptides used for the final MS‐PRM analysis are shown in Table 2.

**TABLE 2:** Description of peptide origin and properties for PRM Validation

Origin (enzyme specificity)	Heavy isotope‐labeled peptides	Labeled residue	RT window min (expected)	Ratio (L/H) (mean ± SD)	Peptidoforms charge state	Relative ion collection target (time limit)
N‐terminal (Specific)	5‐I(R)VTEDENEPIEIPSEDDGTVL	+10 Da	43–88 (47.5)	0.54 ± 0.62	2+	100% (50 ms)
C‐terminal (Specific)	375‐SGSNS(G)AAIGW	+3 Da	30–45 (40.5)	0.43 ± 0.3	1+	100% (50 ms)
Truncation 1 (N‐terminal nonspecific)	175‐(K)LPNSKQSQDEPL	+8 Da	23–33 (25.5)	0.03 ± 0.09	2+ & 3+	100% (50 ms)
Truncation 2 (N‐terminal nonspecific)	266‐SNRQLERSG(R)F	+8 Da	20–30 (22.5)	0.017 ± 0.016	3+	400% (100 ms)

The sequence of chymotrypsin specific or nonspecific (Truncation) heavy isotope‐labeled peptides is given and the starting position within the TDP‐43 amino acid sequence. () indicate heavy isotope‐labeled amino acids (K Lysine, R Arginine, and G Glycine). Given are mean and standard deviation (SD)

Abbreviations: H, heavy peptide; L, light; ms, millisecond; RT, retention time.

### 3.3 C‐terminal TDP‐43 fragments accumulate in the insoluble protein fraction of the ALS cortex

Insoluble protein fractions subjected to MS‐PRM for the absolute quantification of TDP‐43 and CTFs were also assessed by silver staining and immunoblotting for a semi‐quantitative assessment of TDP‐43 pathology characteristics. Silver stains of the insoluble (urea) protein fractions from the same tissue cohort of 16 ALS, 8 PD, 8 AD, and 8 CTL used for PRM were performed at equal volumes to verify the recovery of protein prior to digestion and to confirm depletion of abundant proteins after sequential fractionation (Figure S2). The urea‐enriched insoluble protein fractions were then immunoblotted with an anti‐C‐terminal TDP‐43 antibody that recognizes 20–35 kDa bands of pathologically cleaved TDP‐43 and the full‐length form of TDP‐43. This antibody was chosen to determine the ratio of the C‐terminal lower molecular weight bands to full‐length TDP‐43. In accordance with previous characterizations of the insoluble protein fraction from ALS brains a strong reaction at 25 kDa was detected in motor and prefrontal cortex urea fractions, suggesting that CTFs were present in addition to full‐length TDP‐43 at 43 kDa (1, 5, 6) (Figure 1B). We also observed a 35 kDa band and in some cases a higher molecular smear, likely to represent post‐translationally modified TDP‐43. When we calculated the ratio of CTF levels over full‐length TDP‐43 levels, an increase in the level of CTF‐35 over the level of full‐length TDP‐43 was observed in ALS cortices compared to PD (*p* = 0.04) and an increased level of CTF‐25 in ALS compared to CTL, PD, and AD (*p* = 0.03, *p* = 0.02, and *p* = 0.048) in accordance with the enrichment of CTFs in ALS brain tissue (19). It is noteworthy that CTF‐25 fragments were also detected in the urea fractions of AD cortices, but not significantly different from CTLs. In spinal cord urea fractions the smaller molecular weight bands at 35 kDa and 25 kDa were less prominent and the CTF‐25 to full‐length TDP‐43 ratios unaltered between ALS and CTL, PD, and AD (*p* = 0.08, *p* = 0.17, and *p* = 0.053) (Figure 1C).

### 3.4 Increased C:N‐terminal TDP‐43 peptide ratios in the insoluble protein fraction from motor and prefrontal cortex discriminate ALS from other neurodegenerative diseases

**FIGURE 2:**
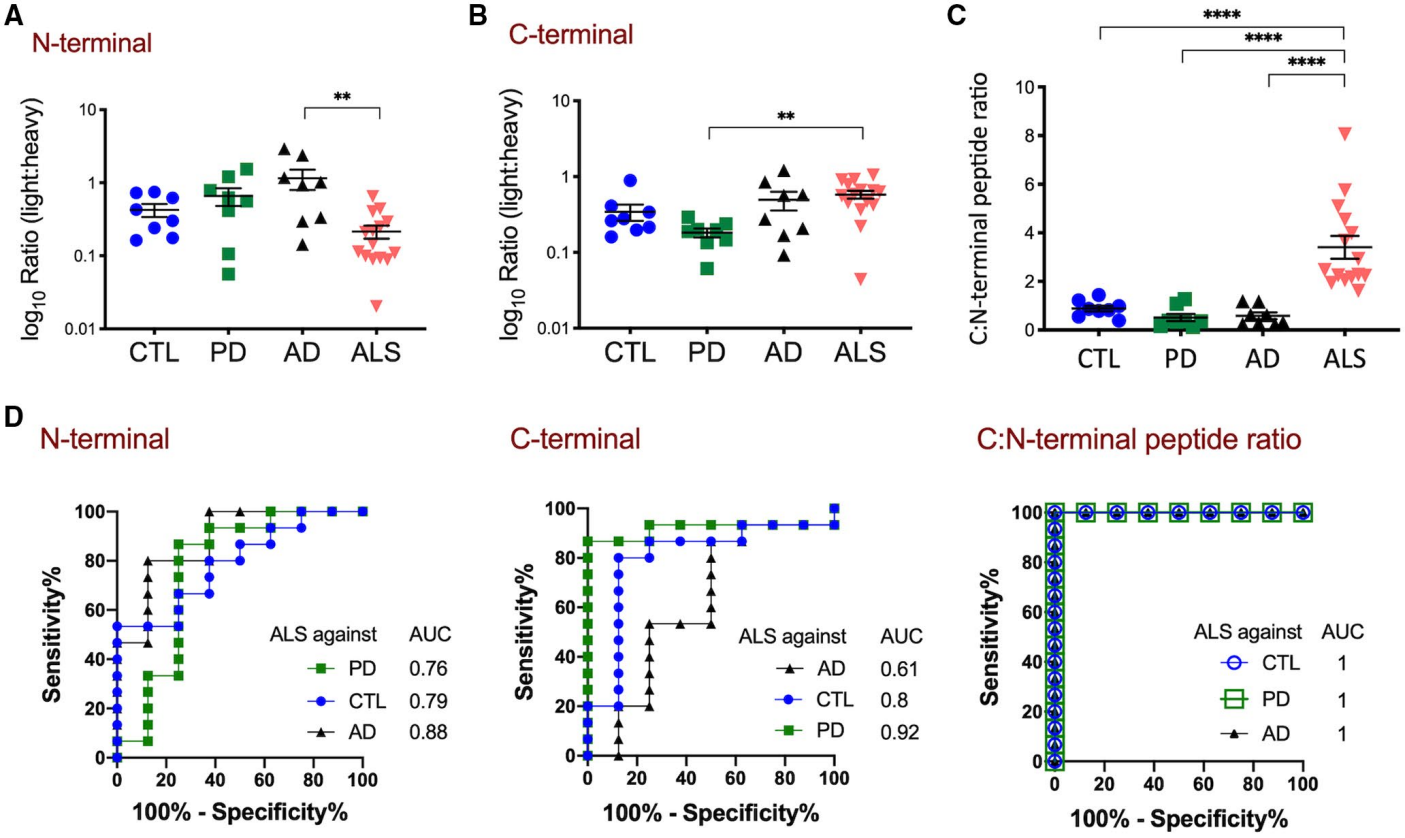
Quantification of C‐ and N‐terminal TDP‐43 peptides in motor and prefrontal cortex urea fractions by MS‐PRM discriminates ALS from other neurodegenerative diseases. (A and B) Shown are absolute abundances of light peptides (log_10_ ratio (light:heavy peptide)) (A) The N‐terminal peptide was decreased in ALS (n = 16) compared to AD (n = 8) (**p = 0.001), but not in ALS compared to CTL (n = 8) and PD (n = 8) (*p* = 0.13 and *p* = 0.051). (B) The C‐terminal peptide was increased in ALS compared to PD (**p = 0.0045), but not in ALS compared to AD and CTL (*p* = 0.7, *p* = 0.29). (C) The calculated C:N‐terminal peptide ratio was increased in ALS compared to AD, PD, and CTL (****p < 0.0001 respectively). One‐way ANOVA with Dunnett’s multiple comparison test. (D) ROC curves of N‐terminal and C‐terminal peptide abundances and the calculated C:N‐terminal peptide ratios for discrimination between ALS and CTL, PD or AD. For comparison, the AUCs are shown

For the absolute quantification of CTFs targeted PRM using heavy isotope‐labeled peptides was used. Quantification of four individual chymotryptic peptides (log_10_ ratio (light peptide:heavy peptide), Figure S3) showed that the N‐terminal peptide was lower in the insoluble protein fraction from ALS cortices compared to AD (*p* = 0.001) (Figure 2A). In contrast, the C‐terminal peptide was increased in ALS compared to PD (*p* = 0.005) (Figure 2B), but unaltered between ALS and CTL or AD (*p* = 0.13 and *p* = 0.81). To investigate if peptide abundances reflect the pathological increase of CTFs in insoluble protein fractions from ALS motor and prefrontal cortex tissue, we calculated the C:N‐terminal peptide ratio. The C:N‐terminal peptide ratio was increased in ALS compared to all other diagnostic groups (*p* = 0.0001, respectively) (Figure 2C). This suggests that the C:N‐terminal ratio is a highly specific marker of ALS representing the enrichment of C‐terminal TDP‐43 protein fragments or a relative lack of N‐terminal TDP‐43 in the insoluble protein fractions, or both.

To determine the potential of using C‐ and N‐terminal peptide quantification as a diagnostic tool to discriminate ALS from other diseases, receiver operating characteristic (ROC) analysis was used (Figure 2D). A N‐terminal peptide abundance <0.3 yielded the optimal discrimination between ALS and AD, with 80% (CI 54.8–93.0) sensitivity and 87.5% (CI 52.9–99.4) specificity. Applying this cut‐off to the CTL group gave a diagnostic sensitivity of 80% (CI 54.8–93.0) and specificity of 62.5% (CI 30.5–86.3), and for the PD group a sensitivity of 73% (CI 48.1–89.1) and specificity of 75% (CI 40.9–95.6). A C‐terminal peptide >0.3 yielded the optimal discrimination between ALS and PD, with a sensitivity of 86.7% (CI 62.1–97.6) and 100% (CI 67.6–100) specificity. Applying this cut‐off to the CTL group gave a diagnostic sensitivity of 86.7% (CI 62.1–97.6) and specificity of 75% (CI 40.9–95.6), and to the AD group, a sensitivity of 86.7% (CI 62.1–97.6) and specificity of 50% (CI 21.5–78.5). A C:N‐terminal peptide ratio >1.5 yielded the optimal discrimination between ALS and CTL, with a sensitivity of 100% (CI 79.6–100) and 100% specificity (CI 67.6–100). Applying this cut‐off to the PD and AD groups gave a diagnostic sensitivity of 93.3% (CI 70.2–99.7) and specificity of 100% (CI 67.6–100).

### 3.5 Truncation peptides for the quantification of pathological cleavage of TDP‐43 in the insoluble protein fraction of ALS and AD motor and prefrontal cortices

**FIGURE 3:**
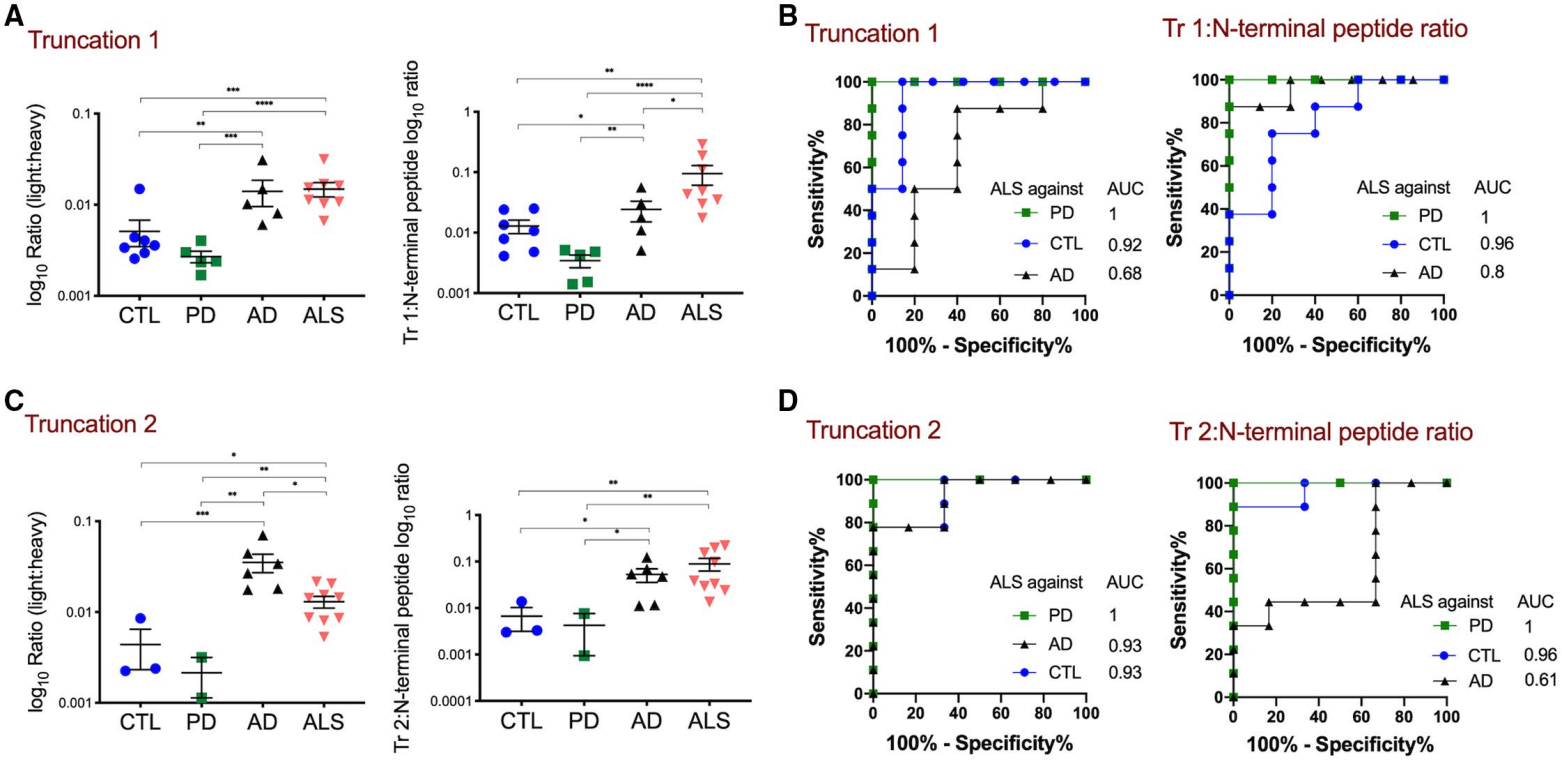
Quantification of truncation site‐specific TDP‐43 peptides by MS‐PRM identifies pathological TDP‐43 processing in motor and prefrontal cortex urea fractions of ALS and AD. (A) and (C) Shown are absolute abundances of light peptides (log_10_ ratio (light:heavy peptide)) (A) The Truncation 1 peptide was increased in ALS (n = 16) compared to PD (n = 8) and CTL (n = 8) (****p < 0.0001, ***p = 0.0008), but not in ALS compared to AD (n = 8) (*p* = 0.94). Truncation 1 was increased in AD compared to PD and CTL (*p* = 0.0004 and 0.007). The Truncation 1: N‐terminal peptide ratio was increased in ALS compared to AD, PD, and CTL (*p = 0.04, ****p < 0.0001 and *p* = 0.001). One‐way ANOVA with Dunnett’s multiple comparison test. (B) ROC curves of the Truncation 1 abundance and the calculated Truncation 1: N‐terminal peptide ratio for discrimination between ALS and CTL, PD or AD. For comparison, the AUCs are shown. (C) The Truncation 2 peptide was increased in ALS compared to PD and CTL (**p < 0.002, *p = 0.01), but decreased compared to AD (*p = 0.01). Truncation 2 was also increased in AD compared to PD and CTL (*p* < 0.0001 and *p* = 0.0001). The Truncation 2: N‐terminal peptide ratio was increased in ALS compared to PD and CTL (**p = 0.004, **p = 0.007), but not AD (*p* = 0.79). One‐way ANOVA with Dunnett’s multiple comparison test. (D) ROC curves of the Truncation 2 abundance and the calculated Truncation 2: N‐terminal peptide ratio for discrimination between ALS and CTL, PD or AD. For comparison, the AUCs are shown

To specifically quantify N‐terminal truncation of TDP‐43, to further proof the pathological truncation of TDP‐43 in smaller CTFs, the truncation site‐specific peptides were measured within the insoluble protein fraction from motor and prefrontal cortex tissue. Quantification of truncation site‐specific peptide 1 (Truncation 1) showed an increase in ALS motor and prefrontal cortex urea fractions compared to PD and CTL (*p* = 0.0008 and *p* < 0.0001, respectively) (Figure 3A). Surprisingly, an increase of Truncation 1 was also measured in AD cases compared to PD and CTL (*p* = 0.0004 and 0.007). Only when calculating the Truncation 1: N‐terminal peptide ratio an increase in ALS compared to AD was observed (*p* = 0.04) caused by the respective higher N‐terminal peptide abundance in AD.

ROC analysis showed that a Truncation 1 peptide abundance >0.005 yielded the optimal discrimination between ALS and PD, with a sensitivity of 100% (CI 67.56%–100.0%) and 100% specificity (CI 56.55%–100.0%) (Figure 3B). Applying this cut‐off to the CTL group gave a sensitivity of 100% (CI 67.6–100) with 85.7% specificity (CI 48.7–99.3). The same cut‐off value applied to the AD group showed a sensitivity of 100% (CI 67.6–100.0), but a low specificity of 20% (CI 1.0–62.5). The Truncation 1: N‐terminal peptide ratio >0.01 achieved a better discrimination between ALS and AD with a sensitivity of 100% (CI 67.6–100.0) and specificity of 40% (CI 7.1–76.9).

Quantification of the new truncation site‐specific peptide 2 (Truncation 2) showed a significant increase in ALS motor and prefrontal cortex urea fractions compared to PD and CTL (*p* = 0.001 and *p* = 0.01, respectively) (Figure 3C), but Truncation 2 was also increased in AD as compared to ALS (*p* = 0.01), PD, and CTL (*p* < 0.0001 and *p* = 0.0001). No significant difference between ALS and AD was observed when calculating the Truncation 2: N‐terminal peptide ratio (*p* = 0.79). According to this, ROC analysis demonstrated a Truncation 2 peptide abundance >0.004 yielded the optimal discrimination between ALS and PD with a sensitivity of 100% (CI 70–100) and 100% specificity (CI 18–100) (Figure 3D). Applying this cut‐off to the CTL group gave a sensitivity of 88.9% (CI 56.5–99.4) and specificity of 67% (CI 11.9–98.3). However, for ALS and AD only a cut‐off value below 0.016 discriminated between both with a sensitivity of 77.8% (CI 45.3–96.1) and specificity of 100% (CI 61.0–100). When the Truncation 2: N‐terminal peptide ratio was calculated discrimination between AD and ALS decreased with an AUC of 0.61. This data clearly demonstrates that biochemically pathological cleavage of TDP‐43 can be measured in both ALS and AD.

**FIGURE 4:**
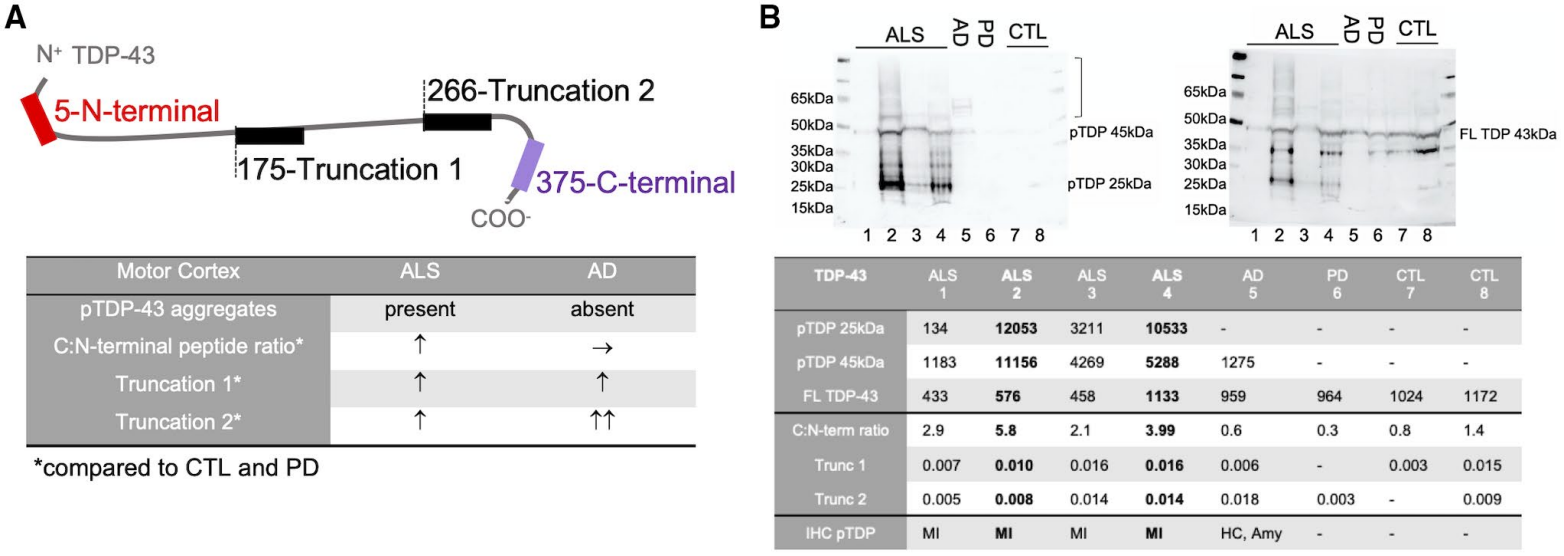
TDP‐43 pathology and peptide quantification in ALS and AD brains. (A) In ALS motor cortices with positive pTDP‐43 pathology an increased C:N‐terminal peptide ratio is present and increased abundance of Truncation 1 and 2 peptides confirms N‐terminal cleavage of TDP‐43. In AD motor cortices with absent pTDP‐43 pathology the C:N‐terminal peptide ratio is normal, but increased Truncation 1 and 2 peptides are indicative of coexisting pathological processing of TDP‐43. (B) Immunoblotting confirms pTDP‐43 immunoreaction (left membrane) at 25 kDa and 45 kDa and a higher molecular smear (]) in ALS motor cortex (MI) with the highest MS‐PRM values for C:N‐terminal ratio, Truncation 1 and 2 and at 45 kDa with a slight higher molecular smear in AD with LATE‐NC in the hippocampus (HC) and Amygdala (Amy) (stage 2). PTDP‐43 is absent in PD and CTL. After stripping FL TDP‐43 at 43 kDa is present in all diagnostic groups (right membrane)

In summary, quantification of the TDP‐43 peptide signature in motor and prefrontal cortex urea fractions showed that in ALS the C:N‐terminal peptide ratio and truncation site‐specific peptides were increased in the presence of TDP‐43 neuropathology. In contrast, in AD motor cortex where TDP‐43 neuropathology is microscopically absent the C:N‐terminal ratio was normal, but truncation site‐specific peptides were similarly increased (Figure 4A).

### 3.6 MS‐PRM quantification of TDP‐43 pathology correlates with phosphorylation of TDP‐43 in brain

To investigate, if an increase of C:N‐terminal peptide ratio and truncation peptides also represent pTDP‐43 pathology we immunoblotted motor cortex urea fractions of ALS and AD cases with the highest MS‐PRM values compared to PD and CTL cases. We observed the highest immunoreaction for pTDP‐43 at 45 and 25 kDa in ALS followed by AD with LATE‐NC, while pTDP‐43 was absent in PD and CTLs with low MS‐PRM values (Figure 4B). As expected, normal full‐length TDP‐43 at 45 kDa was present in all samples.

### 3.7 Detection of biochemical cleavage of TDP‐43 represents co‐morbid TDP‐43 pathology in AD

**FIGURE 5:**
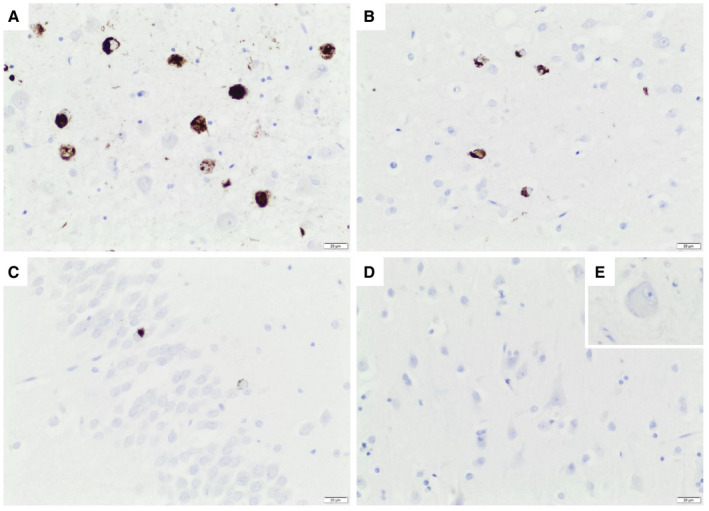
Coexisting LATE neuropathological change in AD brain. Depicted is an AD case with the highest PRM values for Truncation 1 and 2. pTDP‐43 aggregates in (A) amygdala, (B) entorhinal cortex, and (C) hippocampal granule cells according to LATE‐NC Stage 2. No pTDP‐43 aggregates were visible in primary motor cortex (D) and hypoglossal nucleus (E, inset). Scale bar 20 µm

Due to the biochemical evidence of pathological TDP‐43 truncation in AD motor cortex samples using highly sensitive PRM, we sought to investigate if this finding represents the coexistence of LATE‐NC in significant cases of AD. Therefore, we re‐examined our cohort for microscopic evidence of pTDP‐43 pathology. We found evidence of LATE‐NC in six out of eight cases of AD, but not in PD, ALS, or controls confirming the sensitivity of our assay. The principal anatomical distribution of pTDP‐43 in LATE‐NC was different from that typically found in ALS, mainly involving the amygdala, entorhinal cortex, and hippocampus, but not the motor cortex (Figure 5).

### 3.8 C‐terminal TDP‐43 fragments are not increased in the urea fraction of spinal cord

**FIGURE 6:**
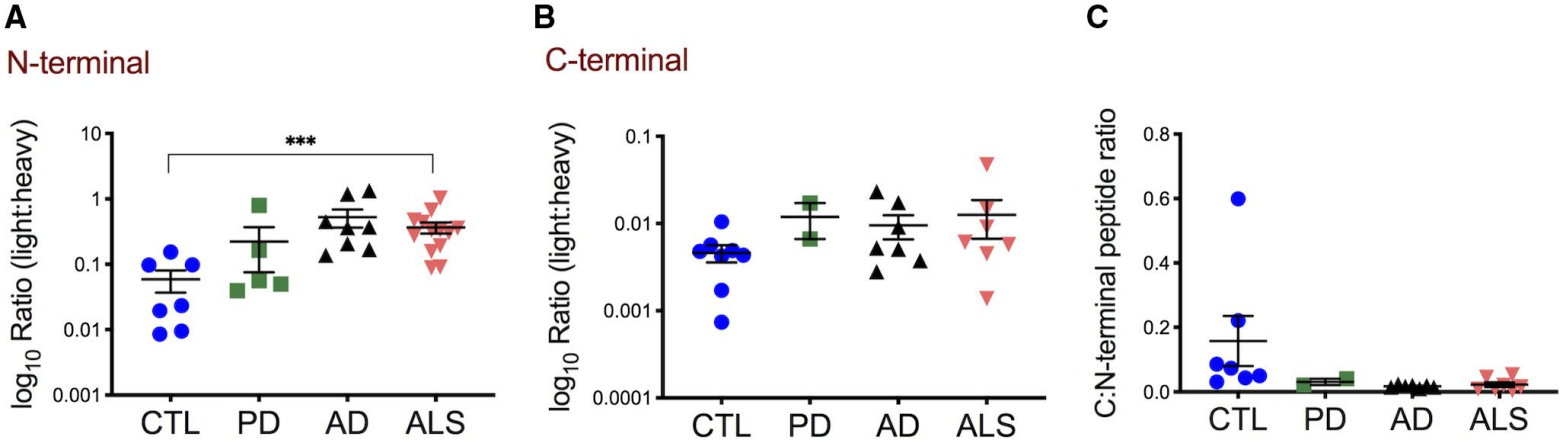
The C to N‐terminal TDP‐43 peptide ratio was not increased in ALS spinal cord urea fractions. (A and B) Shown are absolute abundances of light peptides (log_10_ ratio (light:heavy peptide)) (A) In contrast to motor and prefrontal cortex urea fractions the N‐terminal peptide was increased in ALS compared to CTL (n = 8) (***p = 0.0001), but not in ALS (n = 16) compared to PD (n = 8) and AD (n = 8) (*p* = 0.14 and *p* = 0.88). (B) The C‐terminal peptide was unaltered in ALS compared to all other diagnostic groups (CTL *p* = 0.3, PD and AD *p* = 0.9). (C) The calculated C:N‐terminal peptide ratio was unaltered in ALS compared to CTL (*p* = 0.1), PD or AD (both *p* = 0.9). One‐way ANOVA with Dunnett’s multiple comparison test

To investigate the differences of TDP‐43 pathology in affected regions of ALS, we also investigated case‐matched spinal cord samples. In contrast to data from the cortex, quantification of the N‐terminal light:heavy peptide ratio in spinal cord urea samples showed an increase in ALS compared to CTL (*p* = 0.0001) (Figure 6A). The C‐terminal peptide ratio was unaltered in ALS as compared to CTL (*p* = 0.3), PD, or AD (both *p* = 0.9) (Figure 6B). This suggests the presence of N‐terminal TDP‐43 in spinal cord urea fractions compared to cortex urea fractions, in line with a previous immunohistochemistry analysis, which showed that cytoplasmic inclusions in the spinal cord stain with both a C‐terminal TDP‐43 and N‐terminal TDP‐43 antibody (6). When calculating the C:N‐terminal peptide ratio to assess the proportion of C‐terminal versus full‐length TDP‐43, a reverse pattern compared to cortex ALS urea fractions was observed, with an unaltered C:N‐terminal peptide ratio when compared to CTL (*p* = 0.1) (Figure 6C). Truncation site‐specific peptide 1 was not detected in any of the samples regardless of the diagnostic groups while Truncation 2 was only detected in two CTL, one PD, and one AD sample.

## 4 DISCUSSION

Previous studies measuring TDP‐43 in CSF and serum using antibody‐based methods have revealed inconsistent results due to the nonspecific binding of antibodies to full‐length and pathologically altered TDP‐43 (8, 12). *Post mortem* validation of TDP‐43 pathology currently relies on qualitative rather than absolute quantitative data (20). To overcome this problem, we have applied antibody‐free proteomic discovery analysis for the detection of TDP‐43 and its potentially pathognomonic TDP‐43 peptides in the insoluble protein fraction extracted from ALS brain tissue. A sophisticated targeted proteomic methodology was then developed for the absolute quantification of those peptides across different diagnostic groups and identification of an ALS‐specific peptide signature. This included peptides that were located at the N‐terminus, a peptide region that is also recognized by an antibody specifically detecting nuclear and, therefore, full‐length TDP‐43, and the C‐terminus of TDP‐43 found accumulated in pathological TDP‐43 aggregates in ALS brains (6, 13). In addition, a novel truncation site‐specific peptide with cleavage at amino acid 266, which has the potential to reflect N‐terminal cleavage of CTFs, was identified at 25 kDa by in‐gel digestion. The calculated molecular weight of the CTF generated by the new truncation is only 18 kDa, which either suggests homodimerization of different CTFs or post‐translationally modification of peptides. The first is supported by the detection of other truncation site‐specific peptides within the same in‐gel molecular weight range and also demonstrates low resolution of immunoblots. The pathological basis for the novel truncation site is unclear, as previously described reactions such as calpain‐ or caspase‐ cleavage to generate fragments of the size of 25 kDa and 35 kDa, or cleavage by other peptidases (PeptideCutter), do not occur on the relevant amino acid site (21, 22). A peptidase independent mechanism of asparagine cleavage and protein cross‐linking has been described for long‐lived proteins in aging such as Tau and Amyloid‐ß (23).

Using combined peptide quantification by PRM showed that measuring an increased C:N‐terminal peptide ratio is a sensitive and highly specific marker for ALS TDP‐43 pathology in brain. This is consistent with the accumulation of CTFs in urea fractions of ALS brain tissue as previously assessed by semi‐quantitative measures (1). Our data, quantifying an increase of truncation site‐specific peptides in ALS, also support N‐terminal truncation as the disease‐specific mechanism for C‐terminal TDP‐43 accumulation in the brain. Interestingly, this study also showed that the C:N‐terminal peptide ratio was normal and truncation peptides were mostly absent in urea fractions from ALS spinal cords. This is in line with results from our immunoblots where no significant increase of the CTF‐25 to full‐length TDP‐43 ratio was observed in spinal cords. This also confirmed previous immunohistochemical findings that cytoplasmic TDP‐43 inclusions within the brain stain for C‐ but not N‐terminal TDP‐43, whereas ALS spinal cord TDP‐43 inclusions are positive for both with a predominance of full‐length over C‐terminal TDP‐43 (6, 7). Whether this comes from alternative truncation of TDP‐43, as evidenced by the lack of detection of truncation site‐specific peptides and unaltered CTF‐35 and CTF‐25 to FL TDP‐43 immunoblot ratios in spinal cord, or as a result of different clearance mechanisms between cortical and spinal motor neurons, or neurons and glia, is unclear (24, 25, 26, 27). This finding should prompt further investigation of differential pathological processing of TDP‐43 in relation to different neuroanatomical regions across clinical subtypes of TDP‐43 proteinopathy defined by regional heterogeneity (19, 28, 29).

Our work also revealed a pathological TDP‐43 signature in some cases with the primary neuropathological diagnosis of severe end‐stage AD. These cases had increased truncation peptides, but a normal C:N‐terminal peptide ratio in motor cortex urea fractions. We were able to confirm that those AD cases harbored the recently described LATE‐NC in the amygdala and hippocampus. Interestingly, the pathological TDP‐43 peptide signature was detected in motor cortex, brain areas that are not known to be affected in immunohistochemically determined stages of LATE‐NC. We speculate that this might indicate that mass‐spectrometric detection of truncation of TDP‐43 may represent a biochemical signal of emerging TDP‐43 proteinopathy before visible, compact microscopic aggregates are found. However, a definitive study on this subject will require more extensive multiregional sampling for MS‐PRM of those brain regions affected in LATE‐NC including the middle frontal gyrus. MS‐PRM and combined unbiased quantitative neuropathology across ALS, FTD, and LATE‐NC will be required to comment on the diverse forms of primary and secondary human brain TDP‐43 proteinopathies (30, 31).

A limitation of this study is that the detection of the disease‐specific peptide signature of TDP‐43 by MS‐PRM was in insoluble protein fractions of tissue, where pathological TDP‐43 accumulates. Furthermore, the sensitivity to identify the pathological TDP‐43 peptide signature in whole brain homogenate, where also soluble TDP‐43 is present, still needs to be determined.

In conclusion, we show that the detection of a specific peptide signature enables the absolute quantification of TDP‐43 pathology in the insoluble protein fraction of brain tissue by MS‐PRM. The measurement of pathological CTF accumulation in *post mortem* ALS brain tissue allows accurate discrimination from other neurodegenerative diseases. If such a signature can be detected by MS‐PRM in accessible biofluids such as serum and CSF or extracellular vesicles isolated from CSF, this approach may be ideally suited for the development of a biofluid assay for ALS‐TDP and, potentially, other TDP‐43 proteinopathies.

## CONFLICT OF INTEREST

Professor Martin Turner, Professor Kevin Talbot, Dr. Emily Feneberg, Dr. Elizabeth Gray, Dr. Roman Fischer, Dr. Philip Charles, and Dr Olaf Ansorge have a patent filed for the TDP‐43 sequences described in this work (Patent application No 2004863.3). Professor Benedikt Kessler, Dr. Mattéa Finelli, and Mr Connor Scott have nothing to report.

## AUTHOR CONTRIBUTIONS

Conception and design of the study: RF, EG, BMK, KT, and MRT; Acquisition and analysis of the data: EF, PDC, MJF, CS, OA, and RF; Drafting a significant portion of the manuscript or figures: EF, PDC, MJF, CS, BMK, RF, EG, KT, and MRT; Review and editing: RF, BMK, EG, KT, and MRT; Funding acquisition: MRT, EF, BMK, and PDC; Resources: RF, BMK, OA, and MRT; Supervision: RF, EG, KT, and MRT. All authors critically reviewed the manuscript and approved its submission.

## Supporting information

Supplementary Material
**FIGURE S1** Neuropathology of the study cohort. (A) ALS‐TDP with granular‐, skein‐ and neuritic pTDP‐43 aggregates in the motor cortex (layer three) and nucleus hypoglossus (inset). (B) PD with alpha‐synuclein‐positive Lewy bodies, granular deposits and Lewy neurites inthe substantia nigra (inset: pigmented dopaminergic neuron with Lewy body H&E). (C and D) classical AD with a neuritic beta‐amyloid‐positive plaque (C) and neurofibrillary tangles in the granule cells of the hippocampus (D) (serial sections). Scale bar 20 μm
**FIGURE S2** Silver stains of insoluble and soluble protein fractions. Representative silver stains demonstrate the total protein amount of (A) 0.5 μl of the soluble (TX) protein fractions. (B) 1 μl of the insoluble (urea) protein fractions extracted from cortex and spinal cord *post mortem* tissue of CTL, ALS, PD and AD patients. Depletion of abundant proteins is demonstrated in urea fractions
**FIGURE S3** Skyline export of light and heavy peptides for PRM analysis. Representative Skyline exports shown with Savitzky‐Golay (also known as LOESS) retrieved from a pooled ALS cortex urea fraction sample. The top row represents light peptides and the bottom row heavy isotope‐labelled peptides, where different coloured graphs represent fragment ions. (A) N‐terminal chymotryptic light and heavy peptide. (B) C‐terminal chymotryptic light and heavy peptide. (C) Truncation 1 chymotryptic light and heavy peptide. (D) Truncation 2 chymotryptic light and heavy peptide
**TABLE S1** Detailed characteristics of *post mortem* tissue
**TABLE S2** Detection of TDP‐43 peptides from in‐solution trypsin digestions of cortex urea fractions from ALS and CTL cases
**TABLE S3** Detection of semi‐specific truncation site‐specific TDP‐43 peptides from chymotrypsin in‐gel digestion of cortex urea fractions
**TABLE S4** List of TDP‐43 peptides for heavy isotope‐labelled peptide production

## Data Availability

The data that support the findings of this study are available from the corresponding author upon reasonable request. The mass spectrometry proteomics data have been deposited to the ProteomeXchange Consortium via the PRIDE (32) partner repository with the dataset identifier PXD022186.
