# Supplementary material for: Detection and quantification of novel C‐terminal TDP‐43 fragments in ALS‐TDP
Source: Brain Pathol. 2021 Jan 29;31(4):e12923. doi: 10.1111/bpa.12923 (PMC8412074; doi:10.1111/bpa.12923)
Supplement: Supplementary file 1 — Supplementary Material FIGURE S1 Neuropathology of the study cohort. (A) ALS‐TDP with granular‐, skein‐ and neuritic pTDP‐43 aggregates in the motor cortex (layer three) and nucleus hypoglossus (inset). (B) PD with alpha‐synuclein‐positive Lewy bodies, granular deposits and Lewy neurites inthe substantia nigra (inset: pigmented dopaminergic neuron with Lewy body H&E). (C and D) classical AD with a neuritic beta‐amyloid‐positive plaque (C) and neurofibrillary tangles in the granule cells of the hippocampus (D) (serial sections). Scale bar 20 μm FIGURE S2 Silver stains of insoluble and soluble protein fractions. Representative silver stains demonstrate the total protein amount of (A) 0.5 μl of the soluble (TX) protein fractions. (B) 1 μl of the insoluble (urea) protein fractions extracted from cortex and spinal cord post mortem tissue of CTL, ALS, PD and AD patients. Depletion of abundant proteins is demonstrated in urea fractions FIGURE S3 Skyline export of light and heavy peptides for PRM analysis. Representative Skyline exports shown with Savitzky‐Golay (also known as LOESS) retrieved from a pooled ALS cortex urea fraction sample. The top row represents light peptides and the bottom row heavy isotope‐labelled peptides, where different coloured graphs represent fragment ions. (A) N‐terminal chymotryptic light and heavy peptide. (B) C‐terminal chymotryptic light and heavy peptide. (C) Truncation 1 chymotryptic light and heavy peptide. (D) Truncation 2 chymotryptic light and heavy peptide TABLE S1 Detailed characteristics of post mortem tissue TABLE S2 Detection of TDP‐43 peptides from in‐solution trypsin digestions of cortex urea fractions from ALS and CTL cases TABLE S3 Detection of semi‐specific truncation site‐specific TDP‐43 peptides from chymotrypsin in‐gel digestion of cortex urea fractions TABLE S4 List of TDP‐43 peptides for heavy isotope‐labelled peptide production [file BPA-31-e12923-s001.pdf]

Detection and Quantification of Novel C-terminal TDP-43 Fragments in ALS-TDP

Emily Feneberg[1], Philip D. Charles[2], Mattéa J. Finelli[1], Connor Scott[1], Benedikt M. Kessler[2], Roman Fischer[2], Olaf Ansorge[1], Elizabeth Gray[1], Kevin Talbot[1]*, Martin R. Turner[1]*

[1]Nuffield Department of Clinical Neurosciences, University of Oxford, John Radcliffe Hospital, Oxford, OX3 9DU, UK

[2]Target Discovery Institute, Nuffield Department of Medicine, University of Oxford, NDM Research Building, Old Road Campus, Headington, OX3 7FZ, UK

Corresponding authors: Professor Martin Turner and Professor Kevin Talbot

Address: West Wing Level 6, John Radcliffe Hospital, Oxford, OX39DU, United Kingdom

Phone: 0044 1865 223380

E-mail address: martin.turner@ndcn.ox.ac.uk and kevin.talbot@ndcn.ox.ac.uk

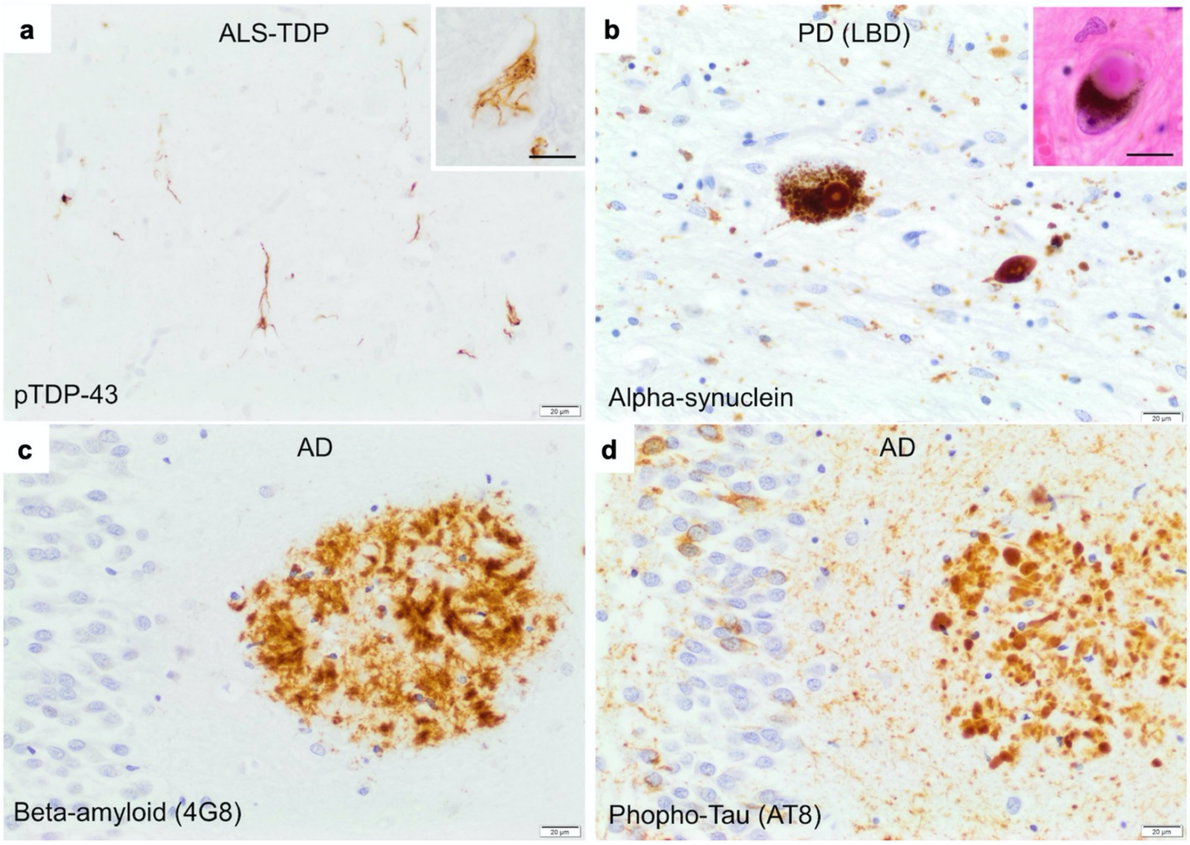

FIGURE S1 Neuropathology of the study cohort

(a) ALS-TDP with granular-, skein- and neuritic pTDP-43 aggregates in the motor cortex (layer three) and nucleus hypoglossus (inset). (**b)** PD with alpha-synuclein-positive Lewy bodies, granular deposits and Lewy neurites in the substantia nigra (inset: pigmented dopaminergic neuron with Lewy body H&E). (**c-d)** classical AD with a neuritic beta-amyloid-positive plaque (c) and neurofibrillary tangles in the granule cells of the hippocampus (d) (serial sections). Scale bar 20µm.

TABLE S1 Detailed characteristics of *post mortem* tissue

Diagnostic group	Gender (f/m)	Age (years)	*Post mortem* delay (hours)	Disease duration (days)	Progression Rate (point/month)	ALSFRS -R	Onset site	El Escorial
ALS	m	57	48	1085	0.45	29	RLL	possible
ALS	f	64	48	591	1.6	21	Bulbar	possible
ALS	m	64	24	629	1.76	18	LUL	probable
ALS	f	59	11	849	0.82	37	Bulbar	definite
ALS	m	76	96	1840	0.72	19	Bulbar	definite
ALS	m	64	48	1312	0.68	20	LUL	definite
ALS	m	62	n/a	1478	0.55	21	LLL	probable
ALS	m	52	72	1861	0.5	17	LLL	definite
ALS	m	76	46	389	n/a	n/a	n/a	n/a
ALS	f	71	n/a	183	n/a	n/a	n/a	n/a
ALS	f	49	57	438	n/a	n/a	Bulbar	n/a
ALS	f	59	11	849	n/a	37	Bulbar	n/a
ALS	m	59	24	1058.5	n/a	n/a	n/a	n/a
ALS	m	86	n/a	n/a	n/a	n/a	n/a	n/a
ALS	f	69	42	n/a	n/a	n/a	n/a	n/a
ALS	m	82	25	3285	n/a	n/a	n/a	n/a
CTL	m	69	24	-	-	-	-	-
CTL	m	66	48	-	-	-	-	-
CTL	f	89	40.5	-	-	-	-	-
CTL	m	63	83.5	-	-	-	-	-
CTL	f	77	70	-	-	-	-	-
CTL	m	63	24	-	-	-	-	-
CTL	f	62	72	-	-	-	-	-
CTL	f	38	41	-	-	-	-	-
PD	m	78	32	4380	-	-	-	-
PD	m	80	24	8395	-	-	-	-
PD	m	79	96	n/a	-	-	-	-
PD	f	80	72	n/a	-	-	-	-
PD	m	79	84	2190	-	-	-	-
PD	m	69	72	n/a	-	-	-	-
PD	f	80	63	3285	-	-	-	-
PD	f	70	24	6205	-	-	-	-
AD	f	87	68	3650	-	-	-	-
AD	f	93	51	n/a	-	-	-	-
AD	f	85	29	2555	-	-	-	-
AD	m	83	120	1460	-	-	-	-
AD	m	85	24	2190	-	-	-	-
AD	m	78	34	3285	-	-	-	-
AD	f	81	24	n/a	-	-	-	-
AD	m	82	65	9490	-	-	-	-

n/a not available, - not applicable, Abbreviations: ALSFRS-R= revised ALS functional rating scale, El Escorial= revised El Escorial clinical certainty criteria, LLL= left lower limb, LUL= left upper limb, RLL= right lower limb, f= female, m= male, ALS= amyotrophic lateral sclerosis, CTL= healthy control, PD= Parkinson Disease, AD= Alzheimer Disease.

TABLE S2 Detection of TDP-43 peptides from in-solution trypsin digestions of cortex urea fractions from ALS and CTL cases

Peptide sequences	-10lgP	RT	m/z	Mass
R.AFAFVTFADDQIAQSLCGEDLIIK.G	81.1	77.47	1336.665	2671.32
R.FGGNPGGFGNQGGFGNSR.G	76.55	40.92	863.8892	1725.761
R.LVEGILHAPDAGWGNLVYVVNYPK.D	64.37	64.89	875.4683	2623.38
R.YRNPVSQCMR.G	63.53	28.24	655.8076	1309.602
R.KMDETDASSAVK.V	62.45	24.38	641.3023	1280.592
K.TSDLIVLGLPWK.T	62.09	66.29	671.3928	1340.77
K.TTEQDLKEYFSTFGEVLMVQVK.K	57.28	72.7	864.7704	2591.283
K.GISVHISNAEPK.H	49.82	31.51	417.8948	1250.662
R.FTEYETQVK.V	48.25	32.27	572.7797	1143.545
**R.VTEDENDEPIEIPSEDDGTVL**LSTVTAQFPGACGLR.Y	44.45	75.74	1292.279	3873.81
R.CTEDMTEDELREFFSQYGDVMDVFIPKPFR.A	33.03	78.44	926.1721	3700.652

Shown are tryptic peptides identified from in-solution digestion. He peptide coverage of TDP-43 for run 1 was 23% CTL and 29% for ALS and 29% and 50% in the second approach. Abbreviations: RT= retention time, m/z= mass/time.

TABLE S3 Detection of semi-specific truncation site-specific TDP-43 peptides from chymotrypsin in-gel digestion of cortex urea fractions

Mol. weight band (kDa)	TDP-43 peptide coverage in %	Peptide sequence and initial amino acid position of TDP-43	-10lgP	RT	m/z	Mass
23	30	133-VQVKKDLKTGHSKGF	52.6	22.39	418.7	1670.9
23		52-RGVRLVEGILHAPDAGWGNLVY	43.6	56.46	598.8	2391.3
25-26		AFVTFADDQIAQSLCGEDLIIKGISVH-264	27.5	69.6	983.2	2946.5
**25-26**	**10**	**266-SNRQLERSGRF**	**30.5**	**23.09**	**450.6**	**1348.7**
25-26		257-ISNAEPKHNSNRQLERSGRF	15.53	22.8	585.8	2339.2

Shown are chymotrypsin semi-specific peptides with the number indicating the enzyme nonspecific amino acid cleavage site at the N- or C-terminal end of the peptide identified from in-gel digested low molecular weight bands of urea cortex fractions. Bold indicates Truncation site-specific peptide 2, which has been used for subsequent MS-PRM analysis. Abbreviations: RT= retention time, m/z= mass/time.

TABLE S4 List of TDP-43 peptides for heavy isotope-labelled peptide production

Protease	Digestion	Sequence	Labelled Residue	Detected
Trypsin	Specific	MSEYI(R)	Arginine (R), +10Da	y
Trypsin	Specific	MSEYI(R)	Arginine (R), +10Da	y
Trypsin	Specific	NPVSQCM(R)	Arginine (R), +10Da	n
Trypsin	Specific	NPVSQCM(R)	Arginine (R), +10Da	n
Trypsin	Specific	LVEGILHAPDAGWGNLVYVVNYP(K)	Lysine (K), +8Da	n
Trypsin	Specific	TSDLIVLGLPW(K)	Lysine (K), +8Da	y
Trypsin	Specific	SS(G)WGM	Glycine (G), +3Da	y
Trypsin	Specific	SS(G)WGM	Glycine (G), +3Da	y
Trypsin	N-Terminal semispecific	EFFSQYGDVMDVFIPKPF(R)*	Arginine (R), +10Da	n
Trypsin	N-Terminal semispecific	GDVMDVFIPKPF(R)*	Lysine (K), +8Da	y
Trypsin	N-Terminal semispecific	GDVMDVFIPKPF(R)*	Lysine (K), +8Da	y
Trypsin	N-Terminal semispecific	FGGNPGGFGNQGGFGNS(R)*	Arginine (R), +10Da	y
Trypsin	C-Terminal semispecific	FGGNP(G)GFGNQGGFGN*	Glycine (G), +3Da	y
Trypsin	N-Terminal semispecific	PGGFGNQGGFGNS(R)*	Arginine (R), +10Da	y
Chymotrypsin	Specific	**I(R)VTEDENDEPIEIPSEDDGTVL**	Arginine (R), +10Da	y
Chymotrypsin	Specific	(R)NPVSQCMRGVRL	Arginine (R), +10Da	n
Chymotrypsin	Specific	HAPDA(G)W	Glycine (G), +3Da	y
Chymotrypsin	Specific	**SGSNS(G)AAIGW**	Glycine (G), +3Da	y
Chymotrypsin	Specific	GSASNA(G)SGSGF	Glycine (G), +3Da	y
Chymotrypsin	Specific	SSMDSKSS(G)W	Glycine (G), +3Da	y
Chymotrypsin	Specific	SSMDSKSS(G)W	Glycine (G), +3Da	y
Chymotrypsin	N-Terminal semispecific	CDC(K)LPNSKQSQDEPL	Lysine (K), +8Da	n
Chymotrypsin	N-Terminal semispecific	**(K)LPNSKQSQDEPL***	Lysine (K), +8Da	y
Chymotrypsin	N-Terminal semispecific	**SNRQLERSG(R)F**	Lysine (K), +8Da	y

Shown are trypsin and chymotrypsin specific and semi-specific heavy isotope-labelled peptides generated for MS-PRM analysis. In exploratory runs peptide detection was assessed. All peptides were analysed for best technical reliability and the final 4 chymotryptic peptides (bold) selected for final PRM analysis. *Peptides previously described by Kametani et al.. Abbreviations: y=yes, n= no

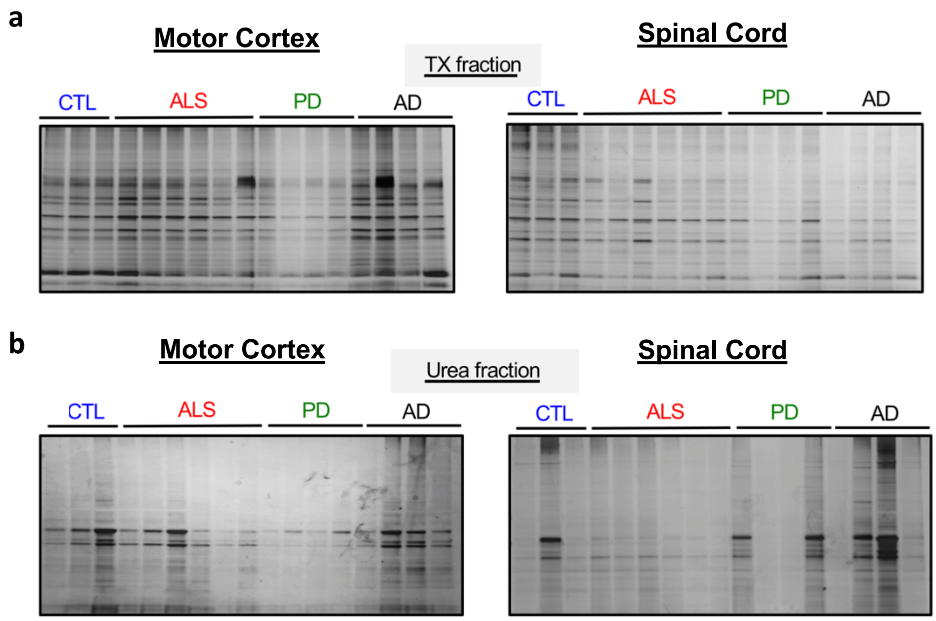

FIGURE S2 Silver stains of insoluble and soluble protein fractions

Representative silver stains demonstrate the total protein amount of **(a)** 0.5µl of the soluble (TX) protein fractions **(b)** 1µl of the insoluble (urea) protein fractions extracted from cortex and spinal cord *post mortem* tissue of CTL, ALS, PD and AD patients. Depletion of abundant proteins is demonstrated in urea fractions.

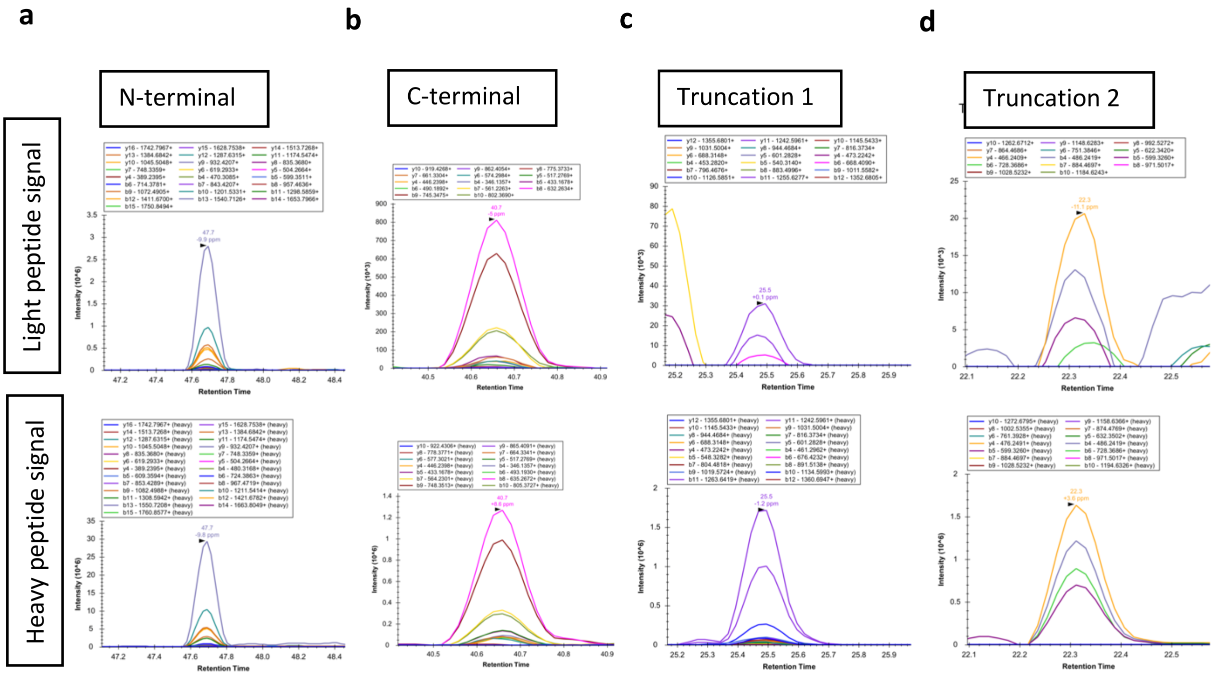

FIGURE S3 Skyline export of light and heavy peptides for PRM analysis

Representative Skyline exports shown with Savitzky-Golay (also known as LOESS) retrieved from a pooled ALS cortex urea fraction sample. The top row represents light peptides and the bottom row heavy isotope-labelled peptides, where different coloured graphs represent fragment ions. **(a)** N-terminal chymotryptic light and heavy peptide. (**b)** C-terminal chymotryptic light and heavy peptide. **(c)** Truncation 1 chymotryptic light and heavy peptide. **(d)** Truncation 2 chymotryptic light and heavy peptide.
